# A Conceptual Framework to Measure Systems’ Performance during Emergency Preparedness Exercises

**DOI:** 10.3390/ijerph110909712

**Published:** 2014-09-17

**Authors:** Elena Savoia, Foluso Agboola, Paul D. Biddinger

**Affiliations:** 1 Department of Biostatistics, Harvard School of Public Health, Boston, MA 02115, USA; 2 Division of Policy Translation and Leadership Development, Harvard School of Public Health, Boston, MA 02115, USA; E-Mails: fagboola@hsph.harvard.edu (F.A.); pbiddinger@partners.org (P.D.B.); 3 Department of Emergency Medicine, Massachusetts General Hospital, Zero Emerson Place, Boston, MA 02114, USA

**Keywords:** emergency preparedness, emergency preparedness exercise, exercise evaluation, measuring preparedness, performance measurement

## Abstract

Large-scale public health emergencies require a sophisticated, coordinated response involving multiple entities to protect health and minimize suffering. However, the rarity of such emergencies presents a barrier to gathering observational data about the effectiveness of the public health response before such events occur. For this reason, public health practitioners increasingly have relied on simulated emergencies, known as “exercises” as proxies to test their emergency capabilities. However, the formal evaluation of performance in these exercises, historically has been inconsistent, and there is little research to describe how data acquired from simulated emergencies actually support conclusions about the quality of the public health emergency response system. Over the past six years, we have designed and evaluated more than seventy public health emergency exercises, collaborating with public health agencies, hospitals and others to test a wide variety of systems and their capabilities. Using the data and experience that we gathered, we have developed a conceptual framework that describes the essential elements necessary to consider when applying performance measurement science to public health emergency exercises. We suggest that this framework may assist practitioners and researchers who wish to better measure performance in exercises and to improve public health emergency preparedness.

## 1. Introduction

Since 2002, the United States has spent well over $20 billion to fund efforts to improve the nation’s health security. Federal, state, territorial, tribal, and local governments, as well as nongovernment organizations and the private sector, have engaged in a wide variety of activities that are intended to improve their abilities to prevent, protect, mitigate, respond to and recover from a myriad of potential health threats. Unfortunately, while there is substantial anecdotal evidence that describes improved performance of the nation’s systems in disaster response, there is little systemic data that would allow critique and comparison of the relative effectiveness of the different programs and interventions. The application of scientifically valid evaluation and quality improvement methodologies to evaluate preparedness efforts was identified as an important strategic objective in the 2012 *National Health Security Strategy* noting that: “developing evaluation methodologies and performance measures is critical for assessing and reporting on progress toward achieving national health security” [1].

However, the rarity of large-scale public health emergencies presents a barrier to gathering observational data about the effectiveness of the public health response before such events occur. With few events, researchers have limited use of statistical methods to test hypotheses and to identify the best predictors of effective response outcomes. For this reason, public health practitioners have relied on simulated emergencies, known as exercises, to routinely test emergency preparedness capabilities and develop improvement plans [2,3,4,5,6,7,8,9]. The formal evaluation of performance in these exercises, however, historically has been inconsistent, and there is little research to describe how data acquired from simulated emergencies actually supports conclusions about the quality of the public health emergency response system.

Over the past six years, we have designed and evaluated more than seventy exercises, collaborating with public health agencies, hospitals and others to test a wide variety of systems and their capabilities [3,4]. The focus of our research has been to study the use of exercises to measure emergency preparedness at the public health system level. We have defined the public health system as the various entities such as governmental agencies, healthcare delivery systems, and private businesses, *etc*. that work individually and together during the response to an emergency. Using the data and experience that we have gathered, we have developed a conceptual framework that describes the essential elements necessary to consider when applying performance measurement science to public health emergency exercises. The scope of this framework is to describe the “conditions” under which performance during an exercise may appropriately be measured and judged to characterize actual readiness. We suggest that this framework may assist practitioners and researchers who wish to better measure performance in exercises and to improve local, national, and global health security.

## 2. Methods

We reviewed exercise materials from more than seventy different public health emergency response exercises that we have designed and evaluated over the past six years, including planning documents, master scenario event lists, evaluator training and briefing materials, participant briefing materials, participant data, after action reports (AARs), and improvement plans, to identify the unique factors in exercise design, execution, observation, data gathering and results analysis that influenced our ability to measure public health system performance during the exercises.

To supplement our own experience and data, we also convened a symposium of experts to discuss what characteristics make an exercise an effective tool to assess emergency preparedness and response. The panel consisted of sixty-one members, and we examined their opinions by the use of nominal group technique (NGT) [10]. Panelists were all practitioners with experience in emergency preparedness exercises and included representatives from federal agencies, state and local health departments (LHDs), large and small jurisdictions, urban and rural areas, and a variety of US regions. The participants were divided into two groups (federal and local government representatives), and each group discussed the characteristics that exercises should have when used to measure preparedness. The groups ranked, discussed and agreed upon the types of financial, organizational, networking, and system-level barriers they each experience when using exercises for performance measurement and agreed upon the “must haves” of a good exercise. More details on the conduction and result of the NGT are summarized below and presented in detail in a previous publication [11].

With the data from our own experience, as well as the data and feedback from our symposium panelists, we developed a conceptual framework to identify each of the essential elements necessary to consider when applying performance measurement science to public health emergency exercises. It is beyond the scope of this manuscript to address the predictive validity of exercise performance versus performance during a real event, we here aim to identify what factors may influence exercise performance measurement during an exercise or series of exercises. We used the definition of “conceptual framework” developed by Jabareen, which is “a network of interlinked concepts that together provide a comprehensive understanding of a phenomenon” [12] and conducted our research to design a framework for practitioners and researchers interested in the use of emergency preparedness exercises to measure the capabilities of a public health system that are required to respond to a threat. We referred to *conceptual framework analysis* [12] to generate, identify and trace the major concepts and to develop key components, each with its own attributes, characteristics, assumptions, limitations, distinct perspectives and specific function within the conceptual framework. The development of the framework is also grounded on data derived from mixed methods such as literature reviews, interviews, in addition to our use of a nominal group technique, and implementation of checklists and surveys during exercises as above. Our approach consists of a continuous interplay between data collection and rigorous analysis with intent to conceptualize it as required by the application of grounded theory [13,14].

## 3. Development and Description of Conceptual Model

Four phases were followed in the development of the conceptual model: (1) Mapping of selected data sources, (2) Reading and categorizing of the selected data, (3) Identifying and naming concepts, (4) Synthetizing and integrating concepts into a conceptual framework.

### 3.1. Phase 1: Mapping The Selected Data Sources

We mapped the spectrum of multidisciplinary literature regarding the use of exercises in emergency preparedness. We identified peer-reviewed articles, books [15], publicly available documents and over 90 AARs (retrospective analysis of the response of an organization to an emergency situation or an exercise). We also reviewed existing exercise performance measurement instruments and related materials made available to the public by the federal government or shared by our state and local partners. [16,17] Another important source of data were interviews conducted with practitioners and scholars from various disciplines engaged as evaluators during our exercises as well as a nominal group techniques (NGT) conducted with practitioners with experience in emergency preparedness exercises selected to represent federal agencies, state and local health departments (LHDs), and various geographical regions.

### 3.2. Phase 2: Reading and Categorizing of the Selected Data

During this phase, we read the selected data and categorized them into type of data (*i.e.*, interviews, literature, and exercise data) and type of research or practice question addressed by the data (*i.e.*, generic use of exercises, measurement methods). The literature review included scientific manuscripts published in peer-reviewed journals and available through Pub-Med, publicly available documents and a systematic review of 90 AARs available through the Lessons Learned Information Sharing (LLIS.gov) describing the response of various U.S. public health systems to the H1N1 pandemic, and three hurricanes: Ike (2008), Gustav (2008) and Katrina (2005). The review of the AARs was performed by two independent reviewers and led to the identification of common challenges faced by various public health systems during the response to major incidents. Measuring performance during an exercise may be assumed as a proxy of performance during a real emergency only when the exercise is a good representation of what could happen during a real incident. Therefore challenges faced during real emergencies, as described in the AARs, must be incorporated in the design of exercise scenarios to make them as realistic as possible. More details on the findings from the review of ARRs can be found in a previous publication [18]. In this manuscript we also derived major conclusions from the conduction of a series of interviews and a NGT. Interviews were performed with 31 practitioners playing the role of “exercise evaluators” with the scope of understanding what type of evaluation forms are most useful to the evaluator for collecting observations during an exercise. Questions were designed to gather feedback of how the questions included in the evaluation forms were formulated, what type of answer options were available and the overall usefulness of the forms. NGT was conducted to depict the characteristics that exercises should have when used to measure preparedness, panelists were divided in two groups led by two facilitators who engaged the panelists in a process to list the “must have” of a good exercise, afterward the two groups of panelists were rejoined as one group and consensus achieved through ranking of the “must haves” reported in the separately, more details on the conduction of the NGT are reported in a previous publication [11].

### 3.3. Phase 3: Identifying and Naming Concepts

In the initial phases of this work, we noted that nearly all organizations use exercises for multiple simultaneous purposes including for training of personnel, gathering data for accountability (*i.e.*, demonstrating satisfactory proficiency to supervisory or regulatory agencies), and/or to identify areas of the plan or response that are in need of significant improvement. When measurement efforts are conducted for accountability purposes, organizations are interested in documenting performance to show their effective stewardship of preparedness resources and their capability to protect the public during emergency situations [19]. Exercise performance measurement focused on “accountability” makes use of data to show how well prepared a health system is, and whether the preparedness of this system is improving overtime. Measuring for accountability purposes involves comparison of performance across jurisdictions or agencies, and thus requires a high-level standardization of methods and measures [20]. On the contrary, when measuring for quality improvement purposes, organizations are more interested in identifying specific flaws and limitations in their plans and systems so that those problems can be addressed before the next emergency occurs. In these instances, the evaluation data gathered and presented are highly granular and specific to the organization and environment. Data used for quality improvement give answers to questions such as “what went well”, “what went wrong”, and “where was the plan inadequate”. As a result of these observations, the first concept identified in the development of our conceptual framework is “*the purpose of the measurement*”.

By the use of a nominal group technique, we brought a group of experts to consensus on what are important features of exercise design and implementation. The group agreed that, during an exercise the absence of senior level players leads to altered decision-making processes, either with respect to the types of decisions or the choice of the appropriate hierarchical level for the decision being made. Similarly, if key response agencies do not participate in the exercise, inaccurate assumptions about their roles and capabilities may be made. Panelists also highlighted the importance of having a detailed, plausible scenario and realistic timeline in the conduct of the exercise to be able to test actual capabilities. Furthermore, panelists stressed the need to have clear and measurable exercise objectives named at the outset of the planning process and a sufficient number of trained and competent external evaluators who are capable of identifying the root causes of the response failures observed. Each of these factors, the quality of the objectives, the availability of key participants, and the ability of those participants that accurately represent their entities’ response, was felt to influence the quality of the exercise, and therefore influence whether the exercise itself was an accurate test of their emergency response capabilities. As a result of these observations a second concept was identified in the development of the conceptual framework: “*the exercise*”.

From the implementation of over seventy exercises conducted by our team, and following interviews with thirty-one exercise evaluators, we realized that, in preparation for an exercise, exercise objectives and performance measures are most commonly selected to assess organizational or system performance. Therefore, the unit of evaluation is at the organizational or system level. During an exercise, however, data are gathered by the observation of individuals’ key actions and decisions forming the bulk of the source of measurement. As a result, there is frequently a discrepancy between the level of data collection (most often individual) and the level of data analysis (organizational or system) when the focus of the evaluation is at the public health system level. As a result of such observations a third concept was identified in the development of the conceptual model: “*the unit*”.

In addition to the work described in this paper analyzing overall exercise evaluation, we have also concurrently examined attributes of the specific measures used in exercise evaluation as well as the attributes of the systems and evaluation forms used to collect performance data. In this work, we have examined and used measures from the Centers for Disease Control (CDC), Office of Assistant Secretary for Preparedness and Response (ASPR), and U.S. Department of Homeland Security (DHS) and many others. Additionally, we have examined and used multiple different forms (“instruments”) for data collection and performance documentation to study the relative merits and shortcomings of each. Through this work, we have even developed our own prototype evaluation forms used by exercise evaluators to gather data on the performance of the system being observed. [3] The evaluation forms that we designed includes a combination of quantitative and qualitative measures, such as checklist of actions that allow evaluators to measure performance against specific observable outcomes (*i.e.*, was a specific task performed or not) and rating scales (*i.e.*, 10 point Likert scale) allowing evaluators to judge how well the task was performed and open-ended questions allowing evaluators to add contextual elements to their assessment, describing root causes of a response failure or recommendations for improvement. The use of any type of evaluation form implies the need to take into account attributes such as reliability, validity, and feasibility. Based on our experience in using multiple types of evaluation forms we identified a fourth concept in the development of the conceptual model: “*the instrument*” defined as the combination of performance measures used during a given exercise and came to the conclusion that multiple types of measures need to be included (checklist, score, open ended questions) when using exercises to measure performance.

### 3.4. Phase 4: Synthetizing and Integrating Concepts into a Conceptual Framework

**Figure 1:**
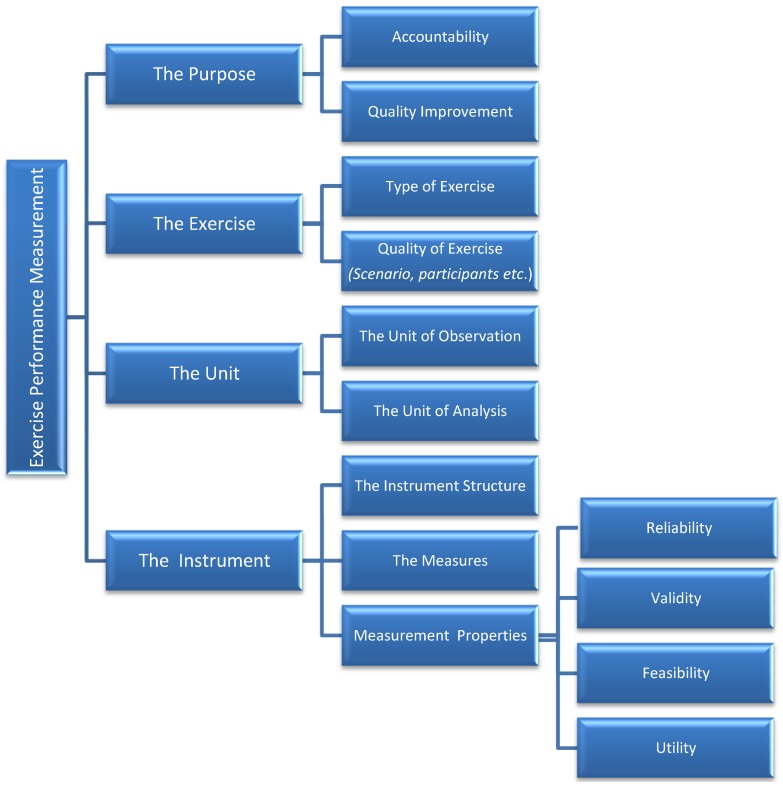
Conceptual model of exercise performance measurement

The concepts and attributes identified above were discussed, grouped and integrated into a conceptual framework depicted in Figure 1. The framework includes four concepts: (1) the purpose of the measurement, (2) the exercise, (3) the unit and (4) the instrument. Measurement of performance by the use of emergency response exercises is done for two main reasons: accountability and quality improvement. Determining the purpose before an exercise helps to identify the best metrics to achieve this purpose. Characteristics that make an exercise an effective tool to assess PHEP are described by the type of exercise (*i.e*., drill, tabletop, functional, full-scale) and its quality (*i.e.*, realism of the scenario, senior level participation, *etc*.). More details on such characteristics can be found in a previous publication [11]. The unit is intended as the level at which data are gathered and analyzed. When the two levels differ, optimal use of exercises to measure performance may be affected. Finally, the use of any measurement approach and instrument comes with measurement properties such as reliability (an estimate of the degree to which an instrument measures its target the same way each time it is used under the same condition with the same subjects), validity (describes how well the conclusions from the measurement process fit with the actual reality) [21], and feasibility (the extent to which the instrument is user-friendly and does not impose excessive burdens on the evaluators). Such properties affect the overall measurement and should be taken into consideration and tested when possible prior to using exercises to measure performance. Conclusions drawn from the exercise response are of limited use if the observational data gathered during the exercise are subjective, inconsistent, or inaccurate.

## 4. Conclusions

It is well recognized that exercises that simulate emergency situations are an essential component of any effective emergency preparedness program. However, despite a strong desire to adequately measure the current state of preparedness efforts by the use of exercises and determine which public health and healthcare capabilities are most in need of investments, the science supporting how best to acquire observational data during exercises, and how best to analyze, compare and aggregate such data is progressing but still limited. There are two main reasons why the science in this area remains inadequate despite a clear need. First, many jurisdictions and agencies are reluctant to publicly share the data that they do gather from their exercises for fear of public criticism. This limits the information available for researchers to study. Second, it is exceedingly uncommon for jurisdictions and agencies to use common evaluation tools or metrics. Evaluation of public health system exercises is frequently done ad hoc, and the data gathered is often narrative in form, rather than quantitative or standardized. This significantly limits researchers’ abilities to aggregate data and draw valid conclusions from the data that is available. Without access to the same metrics and standardized observations collected from multiple exercises across multiple jurisdictions, it is very difficult to study the ways in which exercise performance can best be documented and how it represents true readiness.

In our research, we have sought to develop a conceptual framework within which the preparedness community can begin to address the questions of how to use exercises to best measure preparedness through scientific reasoning. By breaking down the sometimes seemingly impenetrable construct of exercise performance measurement into concepts, we have sought to provide guidance to evaluate the quality of data collected from exercises, and to decipher how such data may be interpreted. Further work is greatly needed into the types of health emergency response capabilities that can be appropriately assessed by each of the different types of exercises. For example, it is very likely that manual tasks are not well measured by tabletop exercises, while longitudinal events, such as pandemics, are poorly studied by full-scale exercises, though there is little data or science to support this conclusion beyond intuition. Also, in addition to continuing to expand the number of performance measures, and testing their measurement properties we believe that it is necessary to urgently develop, test and validate benchmarks of performance for health sector responders. This will only be possible by having multiple responders across the nation using the same measures in multiple exercises, collecting data on those measure in a similar fashion, and repeating this process over time. In the United States, without federal direction and support, however, this will not be possible. Local jurisdictions and agencies will likely always prefer to collect data in their own way and use their own measures of performance until an alternative has clearly been shown to be valid, or until they are required to do otherwise. Yet, it is not possible to prove that an alternative is valid without large-scale data collection and testing. It would be logical for the federal government to dedicate a small amount of resources to support data collection from local public health systems that may validate individual measures of preparedness as well as preparedness benchmarks, and thus scientifically support the measurement of improvement efforts in the nation’s readiness for disaster, to be able to better justify the allocation of funds on preparedness programs.

Our work presents the many limitations to the use of exercises to measure performance. For example, gold standards are non-existent across the instruments used to assess performance as well as external criteria to distinguish high from low performing agencies; therefore, our ability to test the validity of any measurement approach is limited. It is important to be aware of such limitations when policy decisions are made, progress in this research area will lead to a better understanding of how to assess a public health system performance prior to an emergency.
